# Developmental heterochrony and the evolution of autistic perception, cognition and behavior

**DOI:** 10.1186/1741-7015-11-119

**Published:** 2013-05-02

**Authors:** Bernard Crespi

**Affiliations:** 1 Department of Biological Sciences, Simon Fraser University, 8888 University Drive, Burnaby, British Columbia V5A 1S6, Canada

**Keywords:** Autism, Development, Evolution, Heterochrony

## Abstract

**Background:**

Autism is usually conceptualized as a disorder or disease that involves fundamentally abnormal neurodevelopment. In the present work, the hypothesis that a suite of core autism-related traits may commonly represent simple delays or non-completion of typical childhood developmental trajectories is evaluated.

**Discussion:**

A comprehensive review of the literature indicates that, with regard to the four phenotypes of (1) restricted interests and repetitive behavior, (2) short-range and long-range structural and functional brain connectivity, (3) global and local visual perception and processing, and (4) the presence of absolute pitch, the differences between autistic individuals and typically developing individuals closely parallel the differences between younger and older children.

**Summary:**

The results of this study are concordant with a model of ‘developmental heterochrony’, and suggest that evolutionary extension of child development along the human lineage has potentiated and structured genetic risk for autism and the expression of autistic perception, cognition and behavior.

## Background

The autism spectrum is a set of neurodevelopmental conditions characterized and defined by deficits in language and social communication, combined with high expression of restricted interests and repetitive behavior [1,2]. Autism is highly heritable [3], and etiologically heterogeneous, such that a broad range of single-gene, genomic, polygenic and environmental variation has been shown to contribute to the development of a similar, convergent suite of overlapping phenotypes [2,4].

The causes of autism have been studied predominantly using genetic, neurological and psychological conceptualizations and approaches [1,2,4]. The former two approaches have focused on proximate, molecular-genetic, neurodevelopmental, and synaptic causes of autism considered in terms of dysfunctionality and deficits. By contrast, the latter approach commonly involves consideration of both proximate causes and theoretical psychological models of autism-related phenotypes, with autism considered in terms of cognitive or perceptual ‘styles’ or ‘types’, such as weak central coherence [5], high systemizing relative to empathizing [6], or enhanced perceptual function [7,8]. Such styles are atypical but represent extreme manifestations of normally-distributed variation [9], and comprise both relative deficits (mainly in social cognition) and relative strengths (mainly in non-social perception, cognition and task performance).

Evolutionary approaches to the study of autism have been largely restricted to accounts of how phenotypes subject to central deficits in autism, especially language and joint attention, represent uniquely human-evolved or human-elaborated phenotypes (see for example [10,11]). As such, recent human evolutionary trajectories of increasingly complex social cognition have amplified the genetic and environmentally-based scope for losses and alterations of function that differentially impact human social phenotypes, and potentiate risk of autism-related deficits and changes in cognitive functions. This conceptualization of autism, in terms of ultimate, evolutionary causes, has been useful in motivating studies that compare human evolutionary molecular-genetic and phenotypic changes with alterations that typify neurodevelopmental psychiatric conditions, and in comparing autism with other neurodevelopmental conditions such as schizophrenia [11,12]. However, thus far it has provided limited direct utility in proximately-based autism research and development of therapies.

Recent human evolution has involved not just the evolution of enhanced general and social cognition, but also large-scale changes to life history, prominently expressed in extension of childhood, defined as the period from birth to sexual maturity and completion of physical growth [13]. Evolutionary expansion of the human childhood stage, and human adult phenotypes at the endpoint of childhood, have usually been described in terms of neoteny: the retention of juvenile characteristics in adults due to evolutionary changes in rates and timing of development [14,15]. In particular, human physical, neurological and psychological development processes, and their molecular developmental underpinnings, have evolved to take a considerably longer time, presumably through an extended series of genetically-based, heterochronic changes along the human lineage.

As for social cognition considered as a set of adult phenotypes, evolutionary changes generate scope for genetic, epigenetic and environmental alterations in the timing and rates of cognitive development that manifest as neurodevelopmentally-based psychiatric conditions [15,16]. In particular, under this evolutionary-developmental rubric, autism may commonly involve developmental heterochronic shifts in the timing and rates of neurological and psychological development. As such, the suite of phenotypic alterations that characterize autism spectrum conditions would be expected to involve, in part, simple retention of relatively juvenile traits, as extremes of typical temporal sequences of development. The idea of autism as involving reduced developmental rates has been considered previously in general diagnostic terms, but it has yet to be comprehensively evaluated using data from a suite of autism-related phenotypes, to compare ‘autistic’ traits with those of typical child development in an explicitly temporal framework. Moreover, such developmental views as do exist center on deficits and dysfunction, rather than on cognitive differences between younger and older individuals that may be more or less conditionally adaptive as opposed to involving constrained sequences from simpler to more complex [17].

In this article, I evaluate the hypothesis that autism-related traits represent not simply expressions of qualitatively-atypical or ‘pathological’ development, but instead, relatively juvenile phenotypes that have been retained and expressed for longer than is usual in children undergoing typical development. In this context, rather than concentrate on characteristics of autism that represent deficits or absences, I focus on qualitative differences and similarities between autistic individuals and neurotypical individuals of different ages in perception, cognition and behavior. The primary goal of this article is thus to determine the extent to which autism spectrum phenotypes can be regarded as relatively straightforward consequences of shifts in the timing and rates of neurological and psychological development. To the degree that such developmental heterochrony indeed characterizes the autism spectrum, research into causes and therapies for autism might usefully focus more closely on how genetic and environmental variation mediate the rates and timing of child developmental trajectories and milestones, and causes of variability among children in developmentally-structured cognitive styles. A developmental heterochronic structure to autism also implicates recent evolutionary changes along the human lineage in the potentiation and genetic structuring of autistic perception, cognition, and behavior, and risk for autism spectrum conditions.

## Discussion

### Autism and age-related childhood phenotypes

A set of autistic and autism-related phenotypes, derived from models and reviews of the major features, causes, and correlates of autism, was ascertained based on the availability of (1) reasonably well replicated data showing differences between individuals with autism spectrum phenotypes and typically developing individuals, and (2) data that substantiates differences in these same phenotypes between relatively young, and relatively old, typically developing individuals, usually children. The Web of Science and PubMed databases were systematically searched for articles that met these criteria, using a range of related search terms and cited papers within salient articles. Recent review papers were used for especially well-studied or well-documented topics. For topics where directly comparable data were available, patterns observed for autism spectrum conditions were contrasted with those found in the other most thoroughly studied neurodevelopmental condition, schizophrenia, to evaluate patterns of similarity and differences. This article is thus not a systematic review *per se*, but takes a comprehensive approach to surveying the literature with regard to conducting robust tests of the hypothesis proposed.

Four sets of phenotypes were chosen for detailed consideration based on the criteria described above: (1) restricted interests and repetitive behavior, (2) short-range and long-range structural and functional brain connectivity, (3) global and local visual perception and information processing, and (4) the presence of absolute pitch in auditory perception and processing.

### Restricted interests and repetitive behavior

Restricted interests and repetitive behavior represent one of the core diagnostic sets of features for the autism spectrum. This set of behaviors, which follows directly from Kanner’s [18] original descriptions, is defined in *The Diagnostic and Statistical Manual of Mental Disorders*, fifth edition (DSM-5) as involving some combination of stereotyped or repetitive speech or movement, excessive following of routines and resistance to change, highly intense and focused interests, especially high or low sensory reactivity, and fine motor deficits.

This set of behaviors is also characteristic of typical early child development, and is considered to exhibit important stage-specific adaptive functions [19-22]. Thus, Evans *et al*. [19], drawing on the foundational work of Gesell *et al*. [23], described how typically developing 2.5 to 3-year-old children exhibit ‘strong preference for sameness in the environment, repetitive ritualized behavior, rigid likes and dislikes, and sometimes acute sensory perceptual awareness of minute details or imperfections in toys or clothes’, as well as ‘just right’ behavior that includes ‘attention to detail’, ‘heightened awareness of how certain clothes feel’, and ‘ordering of objects in symmetrical patterns’, all of which are notably prevalent among children diagnosed with autism. Such behaviors were reported as highest at ages 2 to 4, with declines thereafter. Glenn *et al*. [21] studied ‘routinized and compulsive-like’ behaviors, also in typically developing children, and found a significant and linear decline between ages 2 and 11.

In the developmental psychology literature addressing autism spectrum conditions, restrictive interests and repetitive behavior, especially motor stereotypies, have traditionally been considered as ‘immature’ behaviors that are normal components of early development but in the autism spectrum have been maintained for longer than normal [22]. Moreover, in children diagnosed with autism spectrum disorder, little atypicality is observed in restrictive interests and repetitive behavior prior to ages 2 or 3 [22].

These findings are in clear agreement with a developmental heterochronic model for autism with regard to restricted interests and repetitive behavior. The causes of the delays *per se* in developmental attenuation of such behaviors have yet to be investigated; restricted interests and repetitive behavior are found most prominently in autistic individuals with relatively low intellectual and language capacity (although restricted interests are also common among autistic individuals with higher intellectual capacity) [22], and higher levels of their expression are correlated with better performance on the embedded figures test, a relative visuospatial strength found in autism [24,25]. However, the strength of the relationship of restricted interests and repetitive behavior with social and language deficits, the other main diagnostic set of features for autism, is moderate to low [26]. To the extent that restricted interests and repetitive behavior exhibit normative adaptive behavioral functions, such as reducing anxiety, decreasing arousal, simplifying complex situations, and fostering a sense of control [20,21], extension of their age-dependent expression may reflect extension of the conditions favoring such behavior. In this context, higher levels of restricted interests and repetitive behavior in autism may be conceptualized in terms of prolonged expression of adaptive, cognitive-behavioral defensive or compensatory functions [27], rather than pathology.

### Short-range and long-range structural and functional brain connectivity

**Figure 1:**
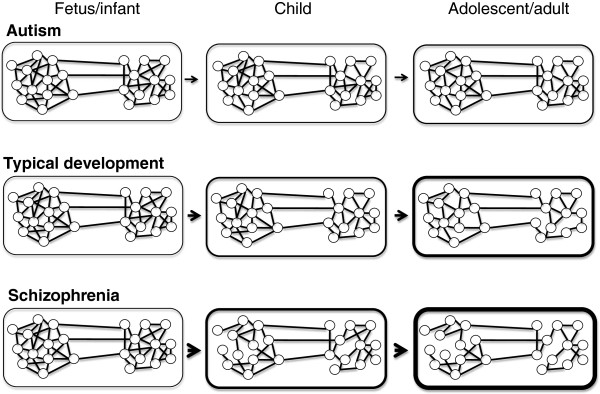
**A simple depiction of the developmental heterochronic model, with regard to changes in short-range relative to long-range structural and functional brain connectivity.** In this specific model, autism involves a slower rate of pruning for short-range connections, and schizophrenia involves a faster rate. Neurodevelopmental variation salient to this process may also involve cortical volume and early short-range connectivity that are greater in autism than in typically developing individuals, and reduced in schizophrenia, which will increase the magnitude of the observed variation in developmental trajectories. Frame thickness is shown as proportional to relative degree of short- range connectivity, and arrow size corresponds to rates of differentiation.

Relatively high levels of short-range functional and structural brain connectivity, concomitant with low levels of long-range connectivity, represent one of the best-replicated and best-supported findings in the study of autism, being reported for a wide range of data sources (magnetic resonance imaging (MRI), diffusion tensor imaging, and electroencephalography (EEG)), analytic methods, and independent sample populations (see for example [28-35]). The causes of relatively reduced long-range connectivity in autism (Figure 1) remain the subject of intense study, but appear to include, among other causes, larger overall brain size, especially in early childhood [28,36], alterations to cortical minicolumns [37], increased dendritic spine density [38], and genetically-based reductions in development of long-range connections [39]. By contrast, increased relative long-range functional connectivity, due to excessive pruning of short-range connections, has been described in schizophrenia [40-43] and auditory verbal hallucinations [44] (Figure 1).

Typical development from infancy to early adulthood involves a robustly characterized shift from relatively short-range to long-range connectivity, in association with early overproduction of neurons and synapses, differential pruning of relatively short-range connections, and increasing myelinization [45-48] (Figure 1). The rates and timing of cortical growth and pruning have been associated with intellectual capacity [49], but connectivity patterns have yet to be systematically evaluated with regard to variation among typically developing individuals in social and language development, or other relatively specific autism-related phenotypes. In the context of evolved risk for autism and its association with larger brain size, it is useful to note that larger brains exhibit relatively low levels of long-range, compared to short-range, connectivity [50,51]. As such, the recent tripling of human brain size has presumably involved selection for developmental-genetic changes that promote increases in relative long-range connectivity [52], which would increase the genetically and epigenetically-based scope for autism spectrum phenotypes, especially in sets of individuals with larger brains, such as males.

Findings on connectivity in autism, and in typical development, concord with a model of heterochronic alterations, with biases towards shorter-range connectivity in autism representing, at least in part, the result of slower or incomplete connectivity-pattern maturation [30]. In principle, behavioral, cognitive and pharmacological therapies that specifically promote the development of relatively longer-range structural and functional cortical connectivity, especially involving default mode and other ‘social brain’ regions, represent productive avenues for future research.

### Local and global perception and processing

The ‘weak central coherence’ theory developed by Francesca Happé posits that autism represents a cognitive ‘style’ characterized by relatively underdeveloped top-down, context-dependent, gestalt, integrated big-picture perception and cognition, and overdevelopment of detail-focused, context-independent, locally-oriented, parts-centered processes [5,53,54]. A central prediction of this theory is a bias in the autism spectrum towards perception and processing of local, relative to global, features of environmental stimuli, as epitomized, for example, by higher performance of autistic individuals, or typically developing individuals with more autism-related psychological traits, on the embedded figures test [55-58].

Overwhelming evidence supports the existence in autism spectrum individuals of biases towards local, compared to global, perception and processing of visual information (reviewed in [59]). However, the degree to which such biases are caused by weaker central coherence, compared to more locally-oriented cognition (with typical central coherence), less interference of global structure with local processing, or enhanced locally-oriented perceptual functions, remains to be established [60-64]. In schizophrenia, relative advantages in global compared to local visual processing have been reported by some studies (see for example [65,66]), but not others (see for example [67,68]), and the specific tasks deployed usually differ from those used in studies of autism. However, performance on the embedded figures test, or comparable figure-ground tests, is reduced in individuals with schizophrenia compared to controls [69-71], and worse performance on this test has also been associated with higher levels of positive schizotypal traits [58] and severity of disorganized and negative schizophrenia symptoms [71,72] (see also [73]).

In typical development, a large suite of recent studies has demonstrated that younger individuals exhibit a local bias in the perception and processing of visual information, which gradually changes to a global bias from about age 4 to adolescence [74-81]. This developmental shift from local to global bias has, moreover, been linked, among typically developing 6-year-olds, with grey matter reductions in right occipital and parietal visuospatial brain regions, such that anatomical maturation through differentially-local pruning appears to ‘fine tune’ the visual cortex for processing global visual information as development proceeds [78]. These findings provide evidence for links of local and global visual processing with short-range and long-range brain connectivity, indicating that such sets of phenotypes subject to developmental heterochronic effects may be causally connected or otherwise interact.

An especially informative specific example of local-global visual processing effects in autistic individuals and typically developing children of different ages comes from studies of visual illusions. A suite of reports has provided evidence that autistic individuals are less susceptible than typically developing individuals to visual illusions [5,82-88], which is consistent with enhanced local and context-independent processing in autism [5,82]. Moreover, decreased susceptibility to visual illusions has been associated with better performance on the embedded figures test [83,89,90] and block design [83,84], as well as a more systemizing cognitive style [91], which indicates that these different measures of autistic perception and cognition reflect overlap and convergence in their neural underpinnings.

Several studies show evidence for increases with age, across childhood, in visual illusion susceptibility [92-94], although some other reports demonstrate an opposite pattern [95]. Such variation, and variation in results among studies of illusions in autism, may be due in part to differences in the specific tests employed, gender-related variation in illusion susceptibility (with males less susceptible; [84,90,96]), and differences in how participants were questioned, regarding whether lines or figures ‘looked’ different, or ‘were’ different [85]. Finally, in contrast to autism, schizophrenia involves overall increases in susceptibility to visual illusions, across multiple studies [97-100], which have been interpreted to indicate ‘excessive spatial contextual effects of the frame’ [100].

These findings generally accord with the developmental heterochronic model, with regard to local and global visual processing. However, more robust tests of the extent to which the local-global processing differences between autistic and typically developing individuals parallel the differences between young and older typically developing children require that the same visual-processing tests be deployed across both groups, and that similar neurophysiological and psychological processes (involving weaker central coherence, stronger local bias, enhanced perceptual function, increased systemizing, or some combination) underlie both the autism-neurotypical and age-related differences. Indeed, partitioning of perceptual-level effects *per se* from effects of enhanced visuospatial processing is required for interpretation specifically in terms of local and global effects. Such studies may usefully proceed by testing for local-global visual processing differences and generally enhanced visuospatial skills between typically developing children who vary in their scores on independent metrics of autistic cognition and behavior.

### Pitch perception

Perception of auditory pitch information may involve absolute pitch (specific pitches) or relative pitch (involving intervals between pairs of consecutive tones). Absolute pitch ability refers to the spontaneous identification of a particular pitch label (for example, middle ‘C’) when exposed to a musical tone, a skill that requires notably increased auditory-perceptual and discrimination skill [101].

The prevalence of absolute pitch ability has been estimated as about 0.01% in typical populations, but between 5% and 11% in autism, and autism involves notably enhanced abilities in pitch discrimination and memory ([102,103]; review in [8]). A higher level of autism-related traits has also been reported in non-clinical individuals with absolute pitch [104,105], and autism spectrum scores have demonstrated significant positive correlation with abilities to correctly identify absolute pitch [105]. In schizophrenia and schizotypy, pitch discrimination and memory abilities are, by comparison, strongly reduced compared to control individuals [106-109], in association with deficits in basic processing of non-auditory as well as auditory sensory stimuli [110,111].

In typically developing individuals, infants perceive absolute pitch more than relative pitch, and show the ability to track ‘extremely fine-grained information’ regarding absolute pitch [112]; by contrast, adults depend primarily on relative pitch [112,113]. In their study Stalinski and Stellenberg [101,114] show that this transition involves a monotonic increase in use of relative pitch information, and a decrease in use of absolute pitch information, during development from ages 5 to 12. The achievement of absolute pitch in typically developing individuals normally requires some level of musical training, which is especially effective at younger ages [115,116]. By contrast, in autistic individuals absolute pitch usually develops before any training in musical skills [8].

Absolute pitch perception shows clear evidence of developmental heterochrony across typically developing and autistic individuals, although it is important to note that pitch discrimination abilities cannot necessarily be equated with use of absolute compared to relative pitch information. Moreover, the degree to which absolute pitch perception in autistic individuals recruits the same brain regions as in typical individuals remains to be established. Of particular interest with regard to developmental transitions in pitch perception is conceptualization of the shift as involving the ‘unlearning’ of absolute pitch perception, because it leads to the generation of overly specific categories of sounds that inhibit learning of higher-level patterns [112,117]. In this general regard, absolute pitch perception in autism may be considered as a specific manifestation of enhanced local, detail-oriented perceptual function [8], which does not complete a typical developmental trajectory.

Autism has also been reported to involve enhancements in other modalities of perception, including, for example, vision [118] and tactile sensitivity [119] (reviews in [7,8]), but these topics have been subject to less extensive investigation.

### Developmental heterochrony, human evolution, and the autism spectrum

Neurodevelopmental conditions such as autism are usually conceptualized in terms of deficits, disorders, disease, genetic abnormalities, and ‘pathological’ expression of maladaptive neurological and behavioral phenotypes. These frameworks for understanding and analyzing autism spectrum conditions can be useful for elucidating proximate mechanisms that underlie the causes of autism, but they tend to implicitly assume that neural and psychological development in autism is fundamentally atypical, due to deviation from normative trajectories that starts in the early stages of brain growth and differentiation.

In this article, I have evaluated an alternative, though not necessarily exclusive, model for the etiology of autism, which is based simply on shifts in the timing and rates of infant and child development. This heterochronic developmental model shows clear concordance with well-replicated patterns in the literature on autism and child development in that across the four domains of (1) restricted interests and repetitive behavior, (2) short-range and long-range functional and structural connectivity, (3) local and global visual perception and processing, and (4) auditory pitch perception and processing, the differences between autistic and typically developing individuals mirror the differences between younger and older typically developing individuals. The latter three domains are also expected to be causally associated, in that shorter-range connectivity may subserve more local perception and processing (see for example [63,120]) as well as finer-grained sensory discrimination, although such associations have yet to be studied in a targeted way.

The aspects of autism evaluated here can thus be explained, in part, as involving developmental delays or non-completion that lead to mismatches of chronological age with perceptual, cognitive and behavioral profiles. Other central phenotypes of autism, such as delay and underdevelopment of language, social reciprocity, joint attention, pretend play, imagination, object identification, and other aspects of cognition and social behavior [1,4,121-123], fit naturally into this simple paradigm. This framework may also be useful in understanding some notable behavioral features of autistic syndromes, such as high levels of positive affect in Angelman and Rett syndromes [124,125]. Finally, the developmental heterochronic model may help to explain the strong male bias found in autism, given that typically developing males tend to undergo slower verbal and social development than typically developing females, and thus may be more vulnerable to alterations that notably delay or impede normative development.

The purpose of a developmental heterochronic conceptualization for the autism spectrum is not to equate it with other diagnostic tools or categories, such as developmental delay, but to demonstrate the nature and ultimate, evolutionary causes of the continuity of autism spectrum phenotypes with typical childhood phenotypes, and to help explain the sources of the otherwise inexplicable constellation of morphological, psychological and behavioral traits first described by Kanner and Asperger. Moreover, to the extent that psychological traits of children represent not just immature, underdeveloped phenotypic stages necessary to reach the adaptive mature target stage, but also in many cases ontogenetically-based, stage-specific adaptations that change in qualitative form as development proceeds [17,126,127], retention of relatively ‘early-development’ traits in autism will necessarily involve some mixture of cognitive enhancements and deficits, as abundantly observed in this literature.

From an evolutionary perspective, genetically-based variation in timing and rates of childhood development is also not unexpected, given that the evolutionary history of human development and life history have followed a similarly structured trajectory of change, towards extension of development in traits ranging from brain gene expression [128,129], to synaptic plasticity [130], synaptic spine development [131] and myelinization [132]. In principle, pathways and sets of genes that underlie growth, neurodevelopment, and synaptic function should thus be expected to overlap between human evolutionary changes (and evolutionary changes earlier in primate and mammalian development) and alterations, as well as segregating variation, that distinguish the autism spectrum from typical development. Such overlaps are reflected in evidence for Darwinian positive selection, and recent human-specific changes in otherwise conserved amino acid positions, in genes such as *AHI1*, *CNTNAP2* and *FOXP2* that have been associated with risk of autism [133-139]. A primary use of evidence from positive selection studies in this regard is that they provide evidence of neurodevelopmental functions for specific haplotypes or amino acid variation, which can direct neurogenetic studies along direct, promising paths. Genes that mediate apparent heterochronic changes and variation have been described from taxa other than humans (see for example [140]), and genetic variation has been associated with the timing of language acquisition traits in autism [141], but the genetic basis of human childhood developmental timing for other traits related to psychiatric conditions has yet to be investigated in any detail. Genetic variation in age-structured gene expression patterns may indeed help to explain the high heritability of autism, as age-related expression adds an additional, temporal dimension to genetic effects on phenotypic variation.

Developmental heterochronic shifts may, of course, proceed in either of two temporal directions. As described above, autism appears to involve delays and non-completion of typical developmental trajectories, which can be considered as extensions of developmental neoteny for some set of neurological systems. By contrast, the evidence described here, as well as previous theoretical considerations of schizophrenia in terms of ‘failures of neoteny’ [15,16], suggest that schizophrenia exhibits elements of premature and accelerated differentiation, the opposite pattern to that observed for autism. Such heterochronic alterations are reflected, for example, in decreased cortical growth and size [142-144], and excessive and relatively early synaptic pruning, neuronal apoptosis and loss of grey matter [145,146], with apparent consequent relative increases in long-range relative to short-range patterns of connectivity, at least for some systems such as the default network [147,148]. As in autism, alterations to childhood and adolescent neurodevelopmental timing and rates are expected to involve decreased performance in some set of psychological traits including language and social cognition, although for fundamentally different reasons [12,149].

Further tests of the developmental heterochronic model, and its contribution to helping explain psychological variation among typically developing individuals as well as in autism, schizophrenia, and related conditions, require integration of information from genetic, developmental, and neurological studies, using analyses that explicitly compare multiple neurodevelopmental conditions in longitudinal frameworks (see for example [146]). The timing of developmental shifts in key neurophysiological systems, such as cortical thickness acceleration and deceleration [49], *N*-methyl-d-aspartate (NMDA) receptor subunit composition [150,151], maturation of primary sensory versus association cortex [152], ratios of excitatory to inhibitory neurotransmission [153-155] and growth and myelinization of long-range connections [29,152], and their genetic bases in such processes as time-dependent production of microRNAs (see for example [156]) and epigenetic regulation of brain maturation by methyl CpG binding protein 2 (MeCP2) [157], should provide relatively strong links between neurodevelopmental timing, its genomic bases, and cognitive outcomes. Such studies will be especially illuminating when conducted in the overall context of recent evolutionary changes in human brain structure and function [139], which are expected to have structured the mechanisms and trajectories of neurodevelopmental shifts. Perhaps most importantly, to the extent that delay or non-completion of cognitive development, rather than primary, pathological alterations, underpin some subset of autism spectrum conditions, autism may be more amenable to treatment than is otherwise believed [158].

## Summary

This article evaluates the hypothesis that some of the major features of autism represent outcomes of shifts in the rate and timing of childhood development, such that traits typical of relatively young individuals are expressed for longer periods, or typical development is not completed. Comprehensive review of the literature indicates that across the four domains of (1) restricted interests and repetitive behavior, (2) short-range relative to long-range functional and structural connectivity, (3) local and global visual perception and processing, and (4) auditory pitch perception and processing, the differences between autistic and typically developing individuals parallel the differences between younger and older typically developing individuals. In contrast to autism, schizophrenia appears to involve developmental heterochronic shifts in the opposite direction, towards accelerated neurodevelopmental differentiation, as reflected, for example, in excessive synaptic pruning and neuronal apoptosis. Given that the evolution of human life history has prominently involved changes in the duration of childhood, these results lend an evolutionary dimension to the analysis of neurodevelopmental psychiatric conditions, and suggest that neurogenetic studies should focus more directly on the causes of variation in rates and timing of childhood neurodevelopmental processes.

## Competing interests

The author confirms that they have no competing interests.

## Pre-publication history

The pre-publication history for this paper can be accessed here:

http://www.biomedcentral.com/1741-7015/11/119/prepub
